# Yorkie is required to restrict the injury responses in planarians

**DOI:** 10.1371/journal.pgen.1006874

**Published:** 2017-07-07

**Authors:** Alexander Y. T. Lin, Bret J. Pearson

**Affiliations:** 1 Hospital for Sick Children, Program in Developmental and Stem Cell Biology, Toronto, ON, Canada; 2 Department of Molecular Genetics, University of Toronto, Toronto, ON, Canada; 3 Ontario Institute for Cancer Research, Toronto, ON, Canada; University of Oxford, UNITED KINGDOM

## Abstract

Regeneration requires the precise integration of cues that initiate proliferation, direct differentiation, and ultimately re-pattern tissues to the proper size and scale. Yet how these processes are integrated with wounding responses remains relatively unknown. The freshwater planarian, *Schmidtea mediterranea*, is an ideal model to study the stereotyped proliferative and transcriptional responses to injury due to its high capacity for regeneration. Here, we characterize the effector of the Hippo signalling cascade, *yorkie*, during planarian regeneration and its role in restricting early injury responses. In *yki(RNAi)* regenerating animals, wound responses are hyper-activated such that both stem cell proliferation and the transcriptional wound response program are heighted and prolonged. Using this observation, we also uncovered novel wound-induced genes by RNAseq that were de-repressed in *yki(RNAi)* animals compared with controls. Additionally, we show that *yki(RNAi)* animals have expanded epidermal and muscle cell populations, which we hypothesize are the increased sources of wound-induced genes. Finally, we show that in *yki(RNAi)* animals, the sensing of the size of an injury by eyes or the pharynx is not appropriate, and the brain, gut, and midline cannot remodel or scale correctly to the size of the regenerating fragment. Taken together, our results suggest that *yki* functions as a key molecule that can integrate multiple aspects of the injury response including proliferation, apoptosis, injury-induced transcription, and patterning.

## Author summary

The planarian displays a remarkable ability to regenerate any tissue from mere fragments of its original size. This high capacity to regenerate is attributed to the abundant population of pluripotent adult stem cells. In response to an injury, such as an amputation, stem cells proliferate and replace the lost tissues de novo (epimorphosis), whereas existing tissue must rescale to the correct proportions in relation to the new fragment size (morphallaxis). Currently, the molecules that control either the responses to injury or the ones that mediate size and scaling are not well understood. For instance, how are the injury responses precisely activated and shut down to ensure regenerating tissues are not under- or overgrown? Here, we find that Yki, the effector of the Hippo signalling cascade, is a critical molecule that influences several injury processes during regeneration. Loss of Yki function in regenerating animals resulted in increased and temporally dysregulated expression of wound-induced genes, proliferation, and apoptosis. Genes that are injury induced were mis-expressed in *yki(RNAi)* animals, which also showed increases in the epidermal and muscle cell populations. Taken together, our findings suggest that the injury responses must be restricted to ensure proper regenerative outcomes of correct scale, and that Yki is a key regulator in these processes.

## Introduction

Regeneration is a growth-controlled program that is observed across the animal kingdom [1]. Neo-natal mice can regrow missing digit tips, salamanders can replace missing limbs, while other organisms, such as hydrazoans and planarians, can regenerate virtually any tissue [1,2,3,4,5]. A common path to successful regeneration is a wounding program that first replaces the missing tissue through proliferation (epimorphosis) and subsequently re-patterns and re-scales the pre-existing tissue to match the new proportions of the animal (morphallaxis) [6,7]. Failure to initiate or cease aspects of either process can result in under- or overgrown tissues, respectively. Yet how proliferation and patterning may be regulated in the correct spatiotemporal manner to determine size and scaling of regenerating tissues remains poorly understood.

The asexual planarian, *Schmidtea mediterranea*, displays a remarkable ability control growth because it can regenerate any missing tissue upon amputation, then rescale the entire animal in proportion to the amount of remaining tissue. Planarian regenerative capacity is derived from its near ubiquitous population of adult stem cells (neoblasts), which are also the only mitotic cells in the animal and at least some of which are pluripotent [8,9,10]. In response to tissue removal, a stereotypical bimodal pattern of proliferation occurs, first at 6 hours post amputation (hpa) and then at 48 hpa [11]. Simultaneously, a generic transcriptional injury response program is expressed with distinct temporal and spatial patterns [12,13]. However, it is unknown how these two processes may be linked and what regulator(s) may determine the onset or decay of responses to injury. As regeneration progresses, the missing tissues are replaced, reintegrated with the prexisiting tissue, and then morphallaxis begins to achieve proper proportions of the regenerated animal [6]. For example, in planarians the WNT polarity gradients must be rescaled to accommodate the new size of the worm [14], and the brain can be dynamically scaled by the combined action of WNT, *notum*, and the hedgehog pathways [15,16]. Therefore, the planarian is a unique model to understand the active processes of growth—proliferation, patterning, and scaling—in an adult regenerative context.

The Hippo pathway is universal regulator of growth control and is a kinase cascade that impinges on the transcriptional co-activator, Yorkie, (in vertebrates: YAP1/2 and paralog TAZ) [17,18]. Constitutive activation of Yki or YAP results in overgrown tissues in flies and vertebrates, suggesting a conserved role in promoting cell division [17,19,20]. However, YAP can be growth-restrictive in highly proliferative and regenerative tissues, such as the mammalian intestine [21]. Indeed, we have previously demonstrated that planarian *yorkie* (*Smed-yki*) is required to restrict stem cell proliferation, yet *yki(RNAi)* animals also fail to regenerate [22]. This conundrum between increased proliferation with an undergrowth phenotype suggests that growth control, patterning, and/or wound-responses are dysregulated, although this remains to be tested. Here, we examine the role of Yki in the known responses to injury, specifically stem cell proliferation and the early transcriptional wound response. In *yki(RNAi)* animals, we demonstrate that the proliferative and transcriptional injury responses are both hyper-activated and temporally prolonged. Despite increased proliferation, stem cells showed no block in differentiation, and surprisingly, produced increased numbers of epidermal and muscle cells. Using an RNA-deep sequencing (RNAseq) time course, we determine that *yki(RNAi)* regenerates have significantly up-regulated known and novel wound-induced genes, many of which have known roles in patterning. We then show that *yki(RNAi)* animals fail to maintain proper size and scaling of the brain, midline, and gut during morphallaxis and have altered kinetics of homeostatic eye replacement. Altogether, this study demonstrates that Yki is a highly pleiotropic yet critical regulator of multiple early wound response processes.

## Results

### yorkie restricts stem cell proliferation during regeneration

**Fig 1:**
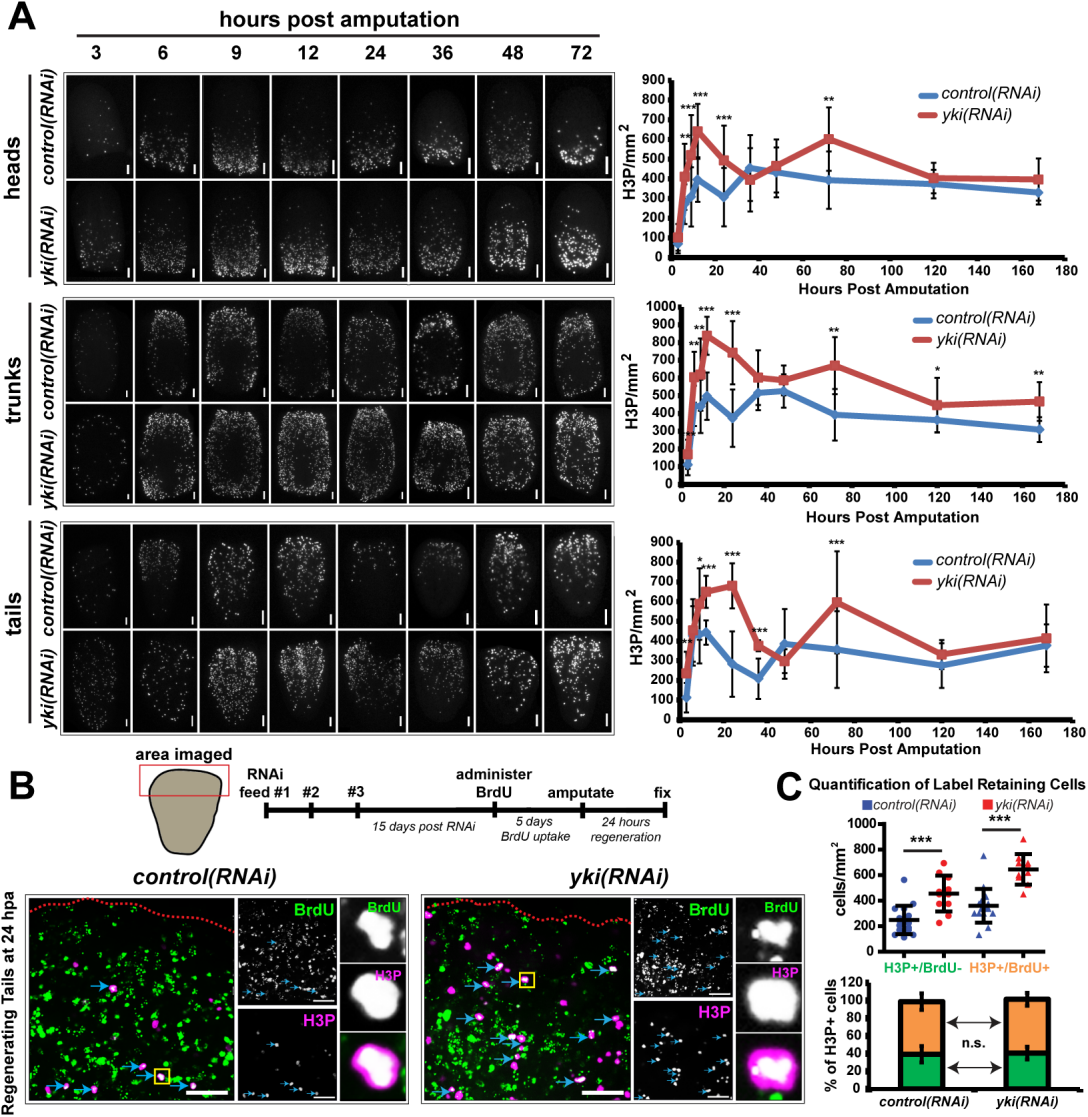
*yki* regulates stem cell proliferation dynamics during regeneration. (A) Representative images for a time course of regenerating fragments (heads, trunks, and tails) stained with mitotic marker, phosphorylated histone 3 (H3P) with quantification on the right (n≥5, N≥2). (B) *yki(RNAi)* does not affect the cell cycle. Top: Timeline and schematic of the pulse-chase experimental details. Bottom: Double positive cells (H3P^+^/BrdU^+^) are marked with blue arrows. Red dotted line is the wound boundary. Yellow box indicates the high magnification of a double positive cell shown in the far right panels. (C) Quantifications of the images in B. Top: A significant increase in the total number of H3P cells is observed in *yki(RNAi)* in both the BrdU^+^ and BrdU^-^ populations. Bottom: No difference is observed (*p* = 0.67) in the percentage/distribution of H3P^+^/BrdU^+^ cells (orange bar) to the total amount of H3P cells in *control(RNAi)* compared to *yki(RNAi)* tail fragments (59±9% and 60±7%, respectively). Error bars are 1 standard deviation. All statistical tests were determined using two-tailed unpaired student’s *t*-tests with *p<0.05, **<*p*<0.01, ***p<0.001, n.s. = not significant. Scale bars indicate 100 μm.

We previously reported that *yki* was required for planarian regeneration, but the underlying causes remained to be elucidated. [22]. Failures in regeneration are often correlated with aberrant stem cell dynamics, including alterations in stem cell proliferation [23,24,25]. Thus, we first tested *yki(RNAi)* regenerating heads, trunks, and tails for defects in proliferation using the G2/M cell-phase marker phosphorylated histone 3 (H3P). In a *control(RNAi)* regeneration time course, we observed that proliferation occurred in a bimodal pattern with the two peaks of proliferation occurring at 9 and 48 hours post amputation (hpa), similar to what has been previously described (Fig 1A) [11]. Surprisingly, in the *yki(RNAi)* time course, the bimodal pattern remained, but the peaks were higher and prolonged. For all 3 fragments, the first wave of proliferation peaked between 12 and 24 hpa, whereas the second wave of proliferation consistently peaked around 72 hpa in *yki(RNAi)* animals (Fig 1A). Proliferation was also sustained at the wound margin in *yki(RNAi)* tail fragments at 72 hpa; whereas, in *control(RNAi)* tails, proliferation predominantly shifted posteriorly away from the wound site by 72 hpa (S1A Fig). From these data, we concluded that similar to intact animals [22], *yki(RNAi)* fragments had increased proliferation based on H3P staining.

Canonical target genes for *Drosophila* Yki are the cell cycle regulator *cyclin E*, and apoptosis inhibitor, *diap1* [17]. Thus, H3P may be an insufficient measure because increased proliferation in *yki(RNAi)* regenerates could be attributed to a change in the cell cycle length. Using BrdU pulse-time-chase experiments, we measured the proportion of label-retaining cells at the wound edge by administering BrdU at 3fd15, amputating worms 5 days later, and fixing and assaying tail fragments at 24 hpa (Fig 1B). The fraction of labeled mitoses at 24 hpa (BrdU^+^/H3P^+^ out of the total H3P^+^) suggested that not only is the proliferation response to injury heightened, but that *yki* does not inhibit S-phase entry or exit. Moreover, the ratio of label retaining cells (H3P^+^/BrdU^+^) to the total amount of H3P cells was not different between *control(RNAi)* and *yki(RNAi)*, 59±9% and 60±7%, respectively, despite higher proliferation in *yki(RNAi)* (Fig 1C). Therefore, the changes in proliferation in *yki(RNAi)* animals cannot be attributed to alterations in the length of the S or G2/M phases individually, although we cannot rule out the possibility that the whole cell cycle is accelerated or that the G1 phase is significantly faster in *yki(RNAi)* fragments. Finally, the increased proliferation was from both sigma- and non-sigma stem cell classes and was also accompanied by increased cell death (S1B and S1C Fig) [26].

Congruent with our previous results, single-cell RNA sequencing (scRNAseq) [13,27] of stem and differentiated tissues demonstrated that *yki* was not enriched in any of the stem cell sub-classes (γ, ζ or σ), and instead, was primarily expressed in differentiated tissues, such as the epidermis, muscle, and gut (S1D Fig) [22]. Interestingly, these same differentiated tissues have recently been shown to be enriched for the expression of wound-induced genes, collectively known as the “transcriptional injury response” [13]. Therefore, we next tested whether changes in the early transcriptional response to wounds could explain either the heightened proliferative response or the failure to regenerate in *yki(RNAi)* fragments.

### yki is required to restrict the transcriptional injury response

**Fig 2:**
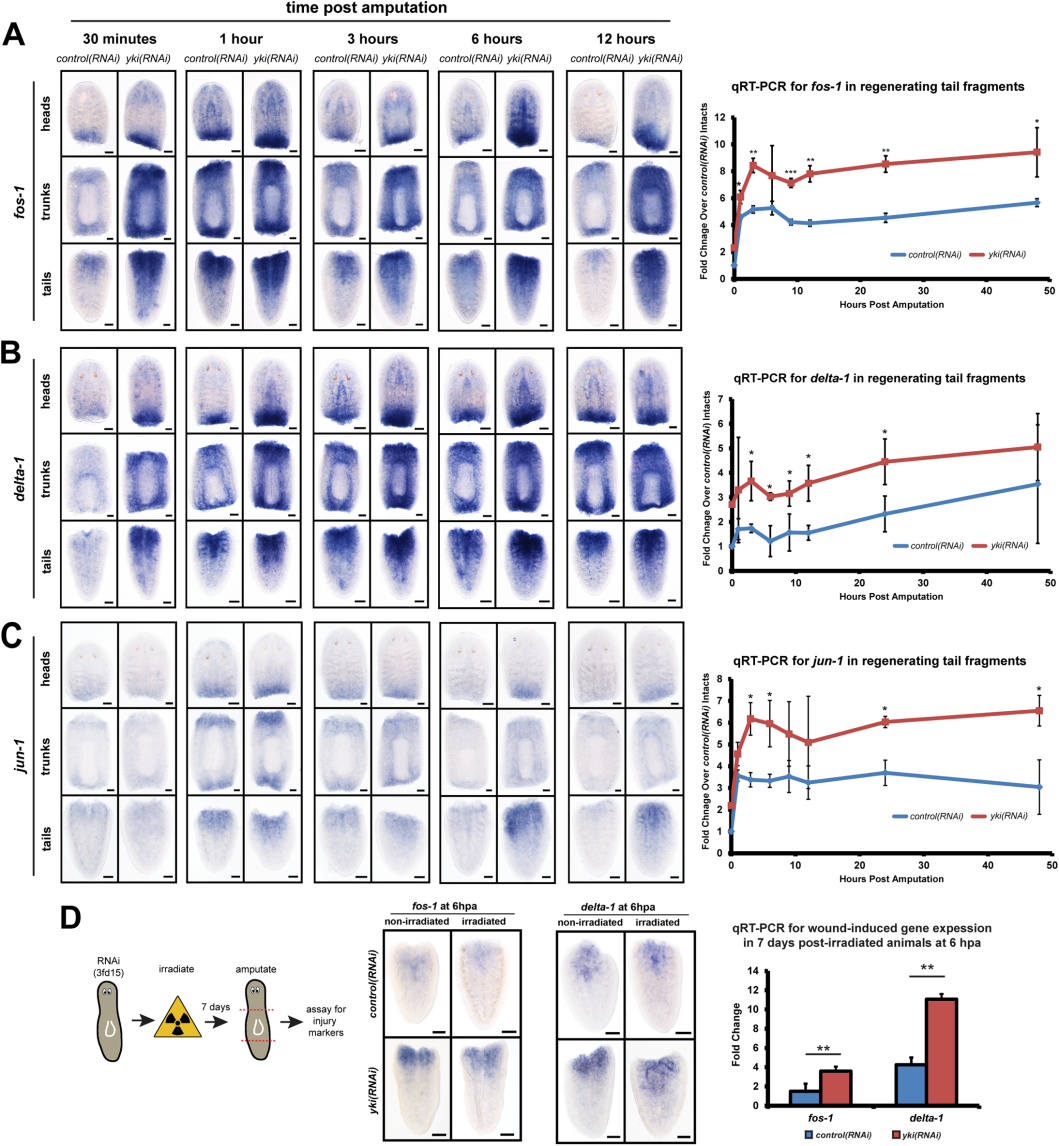
*yki* is required to restrict the transcriptional injury response. Representative WISH time course images of all three regenerating fragments (heads, trunks, and tails) with a corresponding qRT-PCR time course for tail fragments for wound-induced genes *fos-1* (A), *delta-1* (B), and *jun-1* (C). (D) Irradiated worms show the same *fos-1* and *delta-1* response as non-irradiated worms by WISH and qRT-PCR. Each time point had ≥8 animals stained with scale bars denoting 100 μm. qRT-PCR experiments were biologically and technically triplicated, with 1 standard deviation error bars, *p<0.05, **<*p*<0.01, ***p<0.001. All statistical tests were determined using two-tailed unpaired student’s *t*-tests.

In planarians, the transcriptional wounding response program is activated immediately following any type of injury, whether tissue is removed—such as a wedge cut or an amputation—or not, such as an incision or a poke [12]. This response can be subcategorized temporally into two waves of transcription: an immediate “early wave” that peaks in expression by ~6 hpa, and; a subsequent “late wave” that lasts in expression until ~24 hpa [12,13]. In *yki(RNA)*, the early-wave marker *fos-1*, was precociously expressed at 0.5 hpa and was prolonged until 12 hpa (Fig 2A). Similarly, the late marker *delta-1* was also increased and temporally unrestricted in its expression (Fig 2B). The time courses observed by WISH were supported by parallel experiments using qRT-PCR (Fig 2A and 2B). Two other known, wound-induced genes, *jun-1* and *tyrosine-kinase2*, showed similar, upregulated expression in regenerating *yki(RNAi)* head, trunk, and tail fragments (Fig 2C, S2A Fig). Additionally, *yki(RNAi)-*irradiated animals showed no difference in *fos-1* or *delta-1* expression compared to their *yki(RNAi)*-non-irradiated counterparts, suggesting that the increased stem cells/proliferation in *yki(RNAi)* do not contribute to the elevated wound-induced gene expression (Fig 2D). Furthermore, *yki* itself was not injury-induced [13] (https://radiant.wi.mit.edu/app/) and cycloheximde, which blocks protein translation, did not block these transcriptional responses, similar to what has been previously reported (S2B and S2C Fig) [12]. Finally, the known effects of *yki(RNAi)* on the excretory system (edemas) did not alter the proliferative or transcriptional injury responses, demonstrating that the pleiotropic effects of *yki* on the excretory system were independent (S3 Fig) [22]. Due to the heightened transcriptional wound responses in *yki(RNAi)* fragments, we next tested whether transcriptomics of these fragments could be used to discover novel wound-induced transcripts.

### The identification of novel wound-induced genes by RNAseq

The heightened transcriptional injury response in *yki(RNAi)* animals suggested that transcriptome profiling may be able to uncover novel wound-induced genes as well as assay how known wound-induced genes were changing in a more global way. Even though irradiation can cause multiple transcriptional responses in addition to eliminating stem cells [28], we also chose to irradiate animals because *yki(RNAi)* caused an expanded stem cell population and hyper-proliferation, which could significantly alter many non-injury responsive transcripts [22]. Thus, biologically-triplicated RNAseq was performed on 1 day post-irradiated intact animals as well as 1 day post-irradiated regenerating tails at 6, 12, and 24 hpa (see Materials and Methods). Because the transcriptional and proliferative injury responses were not fragment dependent (Figs 1 and 2) and tail fragments had the most penetrant and severe regeneration defects [22], we chose to focus our subsequent analyses on tails.

**Fig 3:**
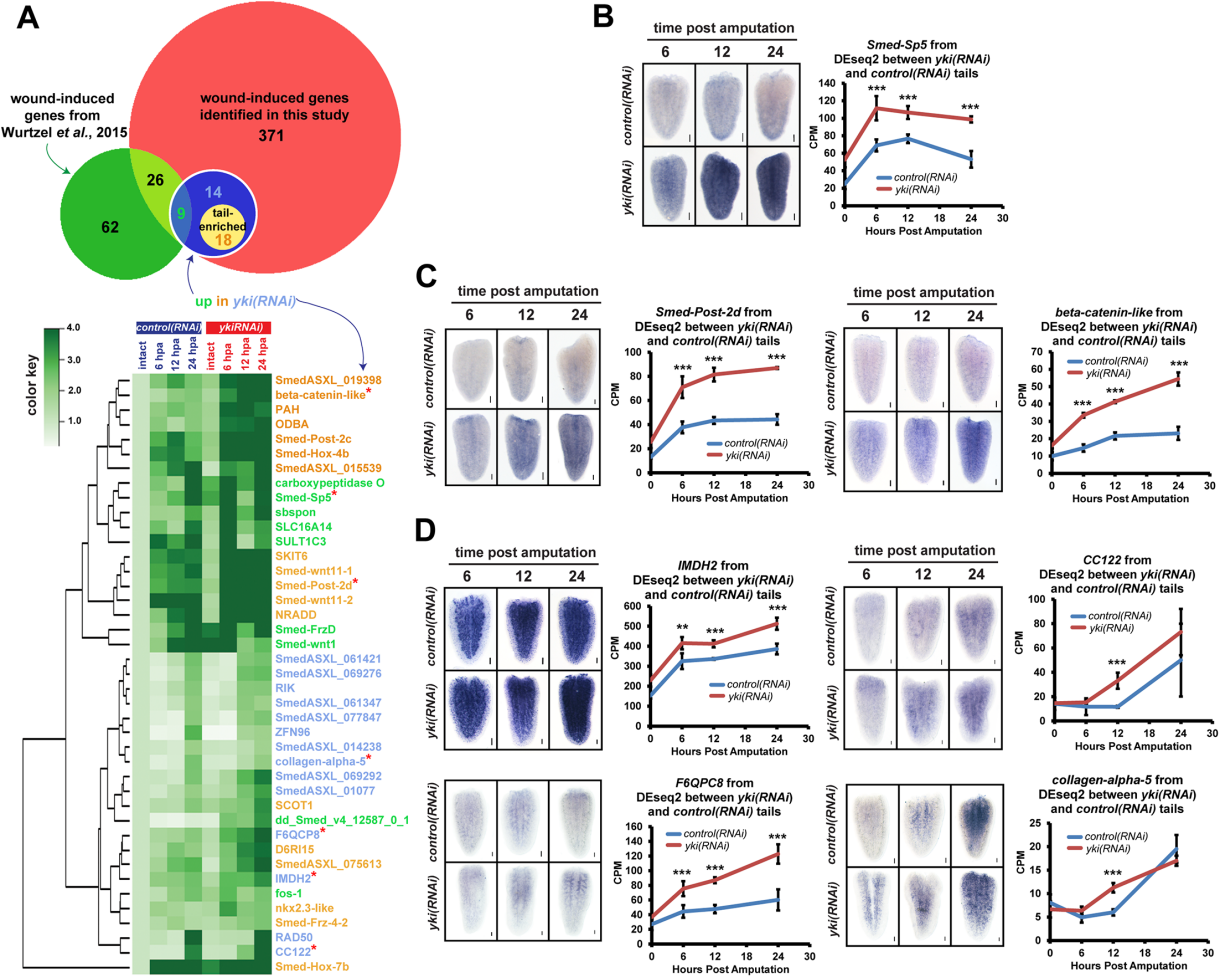
RNAseq analyses of *yki(RNAi)* tail fragments identify novel wound-induced genes. (A) Top: A Venn diagram of wound-induced genes identified in this study (red circle) that were also significantly upregulated in *yki(RNAi)* tail fragments (blue circle). Bottom: A heatmap of the wound-induced genes up in *yki(RNAi)* tails. For relative comparison in the heatmap, *control(RNAi)* tails and all *yki(RNAi)* conditions were compared to *control(RNAi)* intacts for a baseline expression point. Font colors denote: green for previous identification in Wurtzel *et al*. (2015), orange for novel but tail-enriched, or novel and not tail-enriched in blue. Red asterisks indicate transcripts that were validated by WISH. (B) A regeneration time course with representative images of *Smed-Sp5* by WISH (left) and CPM (counts per million) values from RNAseq (right). (C) Representative WISH time course images with corresponding CPM values for novel wound-induced genes that are tail-enriched: *Smed-Post-2d* and *beta-catenin-like*, or (D) not: *IMDH2*, *CC122*, *F6QPC8*, and *collagen-alpha-5*. Significance values were determined by DEseq2 analyses with a FDR<0.05. Error bars are 1 standard deviation. **p<0.01, ***p<0.001. n≥5 for each WISH time point and gene assayed. Scale bars are 100 μm.

In order to determine differential expression between *yki(RNAi)* and *control(RNAi)* tail fragments, we used pair-wise DEseq2, which found 41 transcripts significantly upregulated in the *yki(RNAi)* conditions at any time point (Fig 3A, S1 Table). In general, wound-induced transcripts fell into 3 categories: 1) detected but not validated in previous studies [12,13]; 2) novel from these data, but tail specific [29]; and 3) novel from these data (Fig 3A, S4A and S4B Fig). We subsequently validated several of these transcripts by WISH (Fig 3B–3D; S4C Fig). From these data, we concluded that *yki(RNAi)* animals can not only be used to detect novel wound-induced transcripts, but that wound-responsive transcripts are generally upregulated when *yki* is knocked down (S1 Table). Interestingly, by scRNAseq analyses, many novel candidates had expression in the muscle, while *Smed-Post-2d*, *IMDH2*, and *SmexASXL_061347* also had predominant enrichment in the epidermal (early and late) progenitor populations (S4D Fig) [13]. Therefore, we next examined the epidermal and muscle cell populations for defects in *yki(RNAi)* animals.

### yki(RNAi) animals exhibit higher epidermal density and smaller cell size

**Fig 4:**
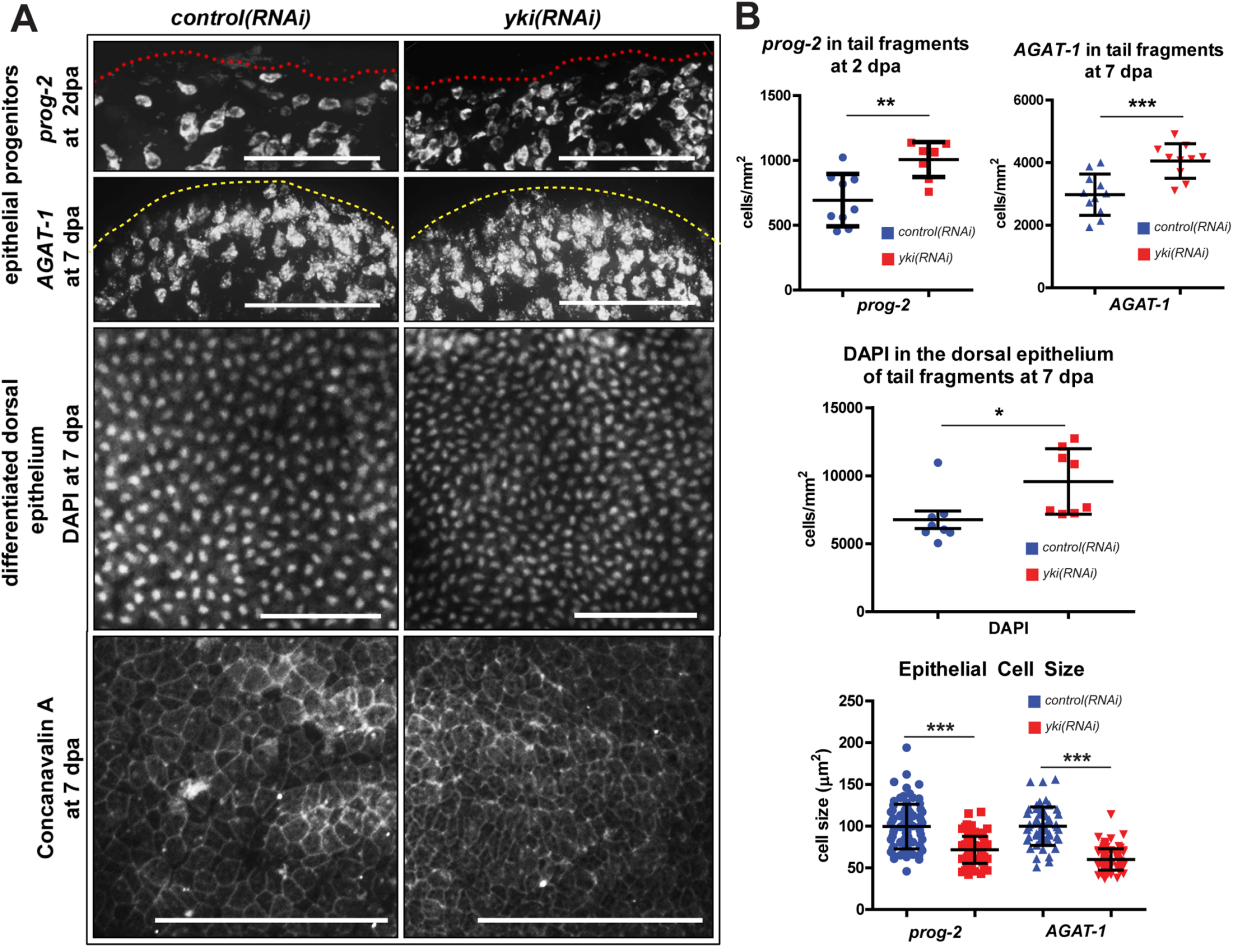
*yki* restricts epidermal density and cell size. (A) Epidermal populations are assayed by WISH for *prog-2* at 2 dpa, *AGAT-1* and DAPI at 7 dpa. Concanavalin A stains for the junctions between epidermal cells. Regenerating tail fragments (n≥8) were assayed for each stain. Red dotted line indicates wound margin, while yellow dashed lines outline the animal. Dorsal views. (B) Histograms of epidermal cell counts and cell sizes by quantifying corresponding images from (A). Each point represents quantification from 1 animal. Error bars are standard deviation. Statistical tests were conducted with two-tailed unpaired student’s *t*-tests with *p<0.05, **p<0.01, ***p<0.001. Scale bars are 100 μm.

The epidermis is enriched for wound-induced genes and has a well-defined lineage that can be readily assayed [12,13,24,26,30,31]. Early epidermal progenitors, marked by *prog-2*, were significantly increased in density in *yki(RNAi)* tails at 2 dpa. Similarly, at 7 dpa, *yki(RNAi)* tails showed significantly more *AGAT-1*^+^ late epidermal progenitors and differentiated dorsal epidermal cells compared to *control(RNAi)* tails (Fig 4A and 4B). This suggested that epidermal differentiation was not blocked in *yki(RNAi)*, and in contrast, *yki* was required to restrict epidermal numbers. The increased epidermal density in *yki(RNAi)* came at the expense of the cell size, and accordingly, the cell size of epidermal cells were also smaller by Concanavalin A staining (Fig 4A and 4B) [32]. Together, these data suggested that the increased stem cell proliferation led to increased epidermal progenitors, and ultimately, increased epidermal density.

### An expanded population of muscle cells and patterning molecules are observed in yki(RNAi) animals

**Fig 5:**
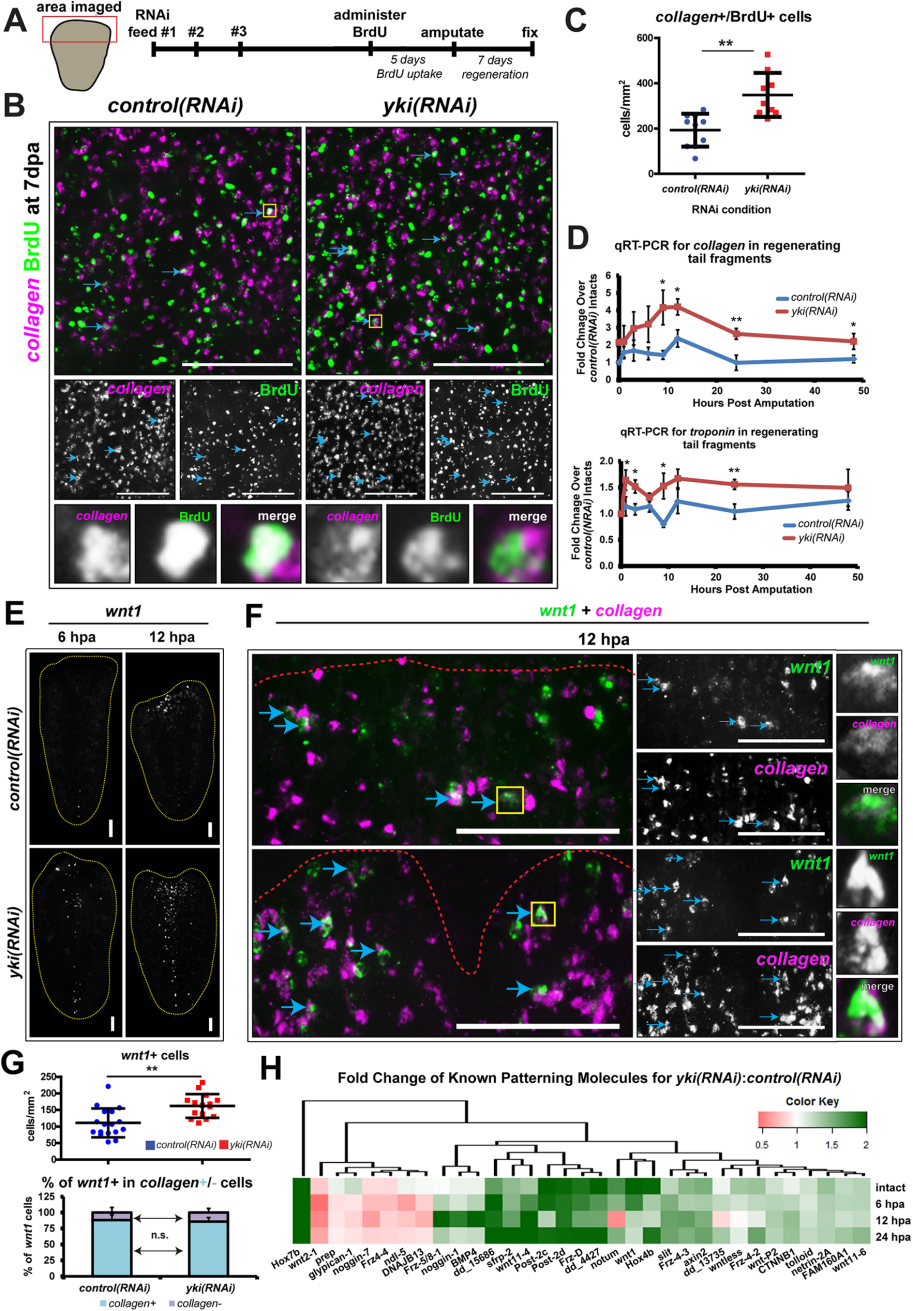
*yki(RNAi)* causes an expansion of the muscle population and mis-expression of patterning molecules, including *wnt1*. (A) A timeline of the BrdU pulse-time-chase experiments into the *collagen*^+^ muscle population. (B) FISH for *collagen* (magenta) and BrdU (green). Double positive cells are denoted with blue arrows. Yellow box indicates the high magnification of the double positive cell in the bottom panels. (C) Double positive cells quantified in a histogram where each point represents 1 animal. (D) qRT-PCR for *collagen* and *troponin* during a regeneration time course. (E) *wnt1* expression in tails at 6 hpa and 12 hpa by WISH. Yellow dotted line outlines the worm fragment. (F) dFISH of *wnt1* (green) and *collagen* (magenta). Double positive cells are denoted with blue arrows. Yellow box indicates the high magnification of the double positive cell in the right panels. (G) Top: A quantification of *wnt1*^+^ cells. Bottom: The percentage of *wnt1*^+^ cells that are also *collagen*^+^ (light blue) or *collagen*^-^ (purple) at 12 hpa. A significant difference in the total number of *wnt1*^+^ cells (top graph) is observed in *yki(RNAi)*, however, the proportion of *wnt1*^+^/*collagen*^-^ to the total number of *wnt1*^+^ cells is unchanged (bottom graph). (H) A heatmap of differentially expressed patterning molecules when comparing *yki(RNAi)* tails to *control(RNAi)* tails. Each stain had ≥8 animals assayed. qRT-PCR experiments were biologically and technically triplicated. Statistical tests were conducted with two-tailed unpaired student’s *t*-tests, with *p<0.05, **p<0.01. Differential gene expression in (H) was conducted with DEseq2. Error bars are 1 standard deviation. Scale bars are 100 μm.

We next focused on the muscle population because it was another cell type that was enriched for many dysregulated wound-induced genes in *yki(RNAi)* (S4D Fig). Moreover, the *collagen*^*+*^ muscle subpopulation is the source of many patterning signals that belong to major signalling cascades such as WNT, BMP, and TGF-β [33,34]. In order to understand the dynamics of the muscle cell population during regeneration, we administered BrdU at 3fd15, amputated worms 5 days later, and subsequently fixed them after 7 days of regeneration (Fig 5A). In *yki(RNAi)* animals, an increased number of *collagen*^+^/BrdU^+^ cells was observed compared to controls (Fig 5B and 5C). Accordingly, we found that *collagen* and another muscle-specific gene, *troponin*, were significantly upregulated immediately after injury in *yki(RNAi)* tail fragments by qRT-PCR (Fig 5D). Thus, the increased muscle cell population could also explain the heightened transcriptional wound response in *yki(RNAi)* (see Discussion).

To test whether the overproduction of muscle cells in *yki(RNAi)* was attributed to increased wound-induced gene expression, we examined *wnt1*. Injury induces *wnt1* expression, which is also a polarity determinant that has >90% co-localization within the *collagen*^+^ muscle cell population [34,35]. In *yki(RNAi)* animals at 6 and 12 hpa, significantly more *wnt1*^+^ cells were observed along the tail midline, but also ectopically at the wound margin (Fig 5E). These ectopic *wnt1*^+^ cells were predominantly *collagen*^+^ with no significant difference in the percentage or distribution of *wnt1*^+^/*collagen*^-^ cells in *yki(RNAi)* as compared to *control(RNAi)* (14.2±3.9% and 11.8±8.1%, respectively; *p* = 0.39) (Fig 5F and 5G). Therefore, *wnt1*^*+*^ was not ectopically expressed in a different cell type and suggested that the expanded muscle population can contribute to the increase in wound-induced gene expression.

Many non-wound-induced body patterning molecules are also known to be highly expressed in *collagen*^*+*^ cells, including the HOXs and FGFs [33,34]. With the increased muscle population in *yki(RNAi)* animals, we tested whether other wound and non-wound induced patterning molecules were aberrantly expressed. Using the same RNAseq paradigm discussed above (Fig 3A), we compared *yki(RNAi)* tails to *control(RNAi)* tails at matched time points and found 33 patterning genes that were significantly dysregulated (Fig 5H, S2 Table). A high proportion of these genes were associated with WNT signalling—a key determinant in anterior-posterior identity—which was expected because *yki(RNAi)* tails do not regenerate their anterior [22]. However, the anteriorly-expressed WNT signalling antagonists, *notum* and *sfrp-2* were still expressed in *yki(RNAi)* tails (Fig 5H, S5A and S5B Fig) [36]. Thus, the *yki(RNAi)* regenerative defects were not simply a failure to express anterior-specification genes. Indeed, the changes in patterning molecules were not limited to the WNTs, but also included genes associated with patterning the midline such as *netrin-2A* and *slit* (S5C and S5D Fig), or the dorsal-ventral axis such as *bmp*, *tolloid-1*, *noggin-1*, and *-7* (Fig 5H). Alterations in expression of these signalling pathways can affect regeneration with improperly sized organs, thus, we next tested whether *yki(RNAi)* animals had defects in organ scaling [16,33,37].

### yki(RNAi) animals exhibit defects in scaling and morphallaxis

**Fig 6:**
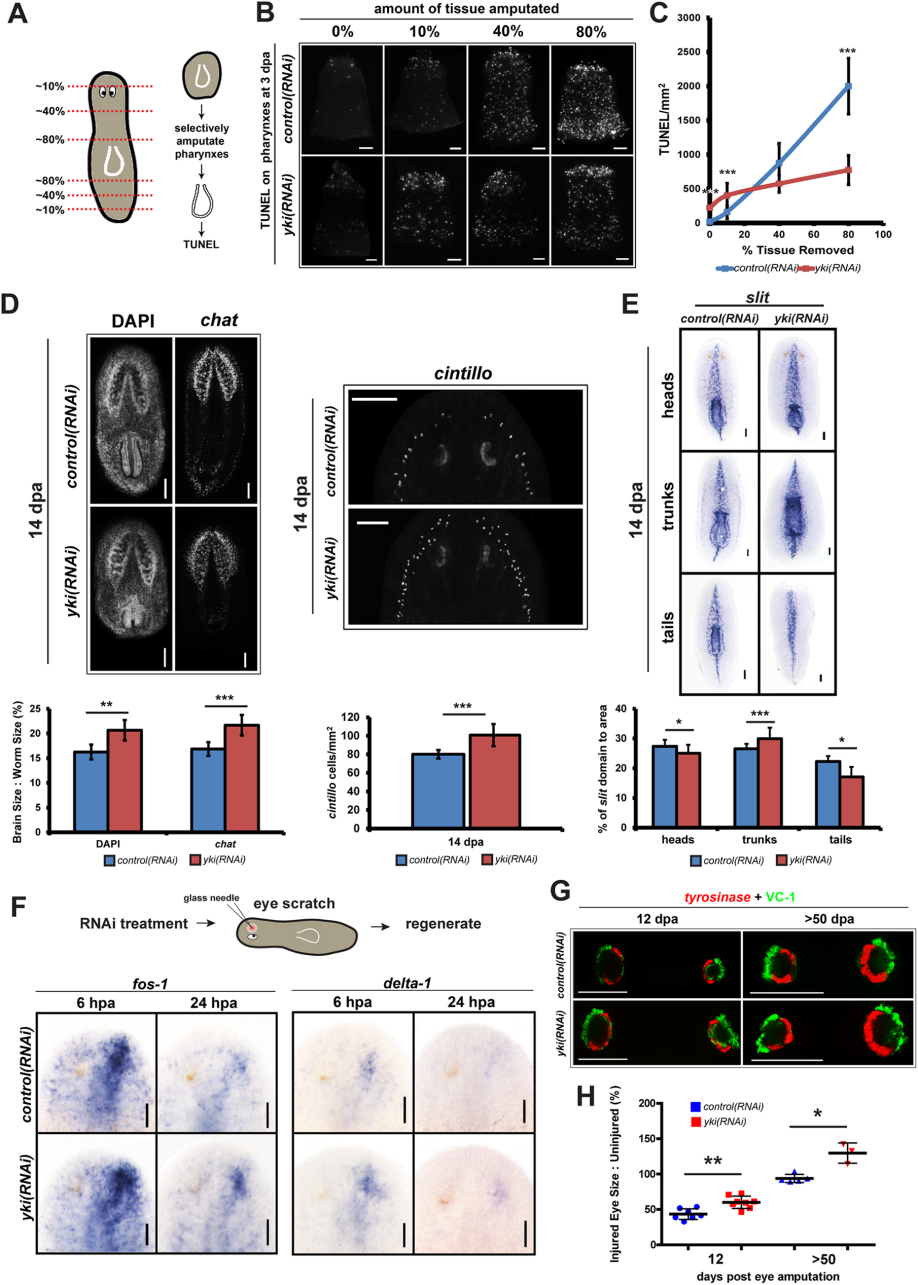
*yki* regulates scaling and morphallaxis. (A) Pharynxes amputated from animals with selective tissue removed can be assayed for cell death as a size sensing mechanism. A schematic of the regeneration series with various percentages of tissue removed with subsequent experimental steps outlined. (B) Amputated pharynxes (n≥9 at each amputation condition) assayed for TUNEL. (C) Quantification of TUNEL stained pharynxes from (A). (D) Top: Head fragments from *control(RNAi)* and *yki(RNAi)* worms were assayed for brain scale and morphology using DAPI (n≥9), *chat* (n≥7), and *cintillo* (n≥8) stains at 14 dpa. Bottom: The ratio of brain size to worm size expressed as a percentage and quantified from (D) and the quantification of the number of *cintillo*^+^ cells to worm size from (D). (E) Regenerating fragments at 14 dpa stained for *slit*. *yki(RNAi)* animals show an expanded *slit* gradient that fails to rescale to appropriate proportions with quantification below (n≥6 for each fragment). (F-H) *yki(RNAi)* animals fail to maintain proper scale and transcriptional injury response dynamics in an eye injury model. (F) Top: schematic of eye scratch experiments. Only right eyes were injured while the left contralateral ones were uninjured. Bottom: *fos-1* and *delta-1* are augmented and prolonged in expression in *yki(RNAi)* animals (n≥5 for each stain and timepoint). (G) Representative animals from the eye scratch experiments at 12 and >50 dpa were assayed for *tyrosinase* (red) and VC-1 (green). (H) A quantification of the regenerating eye size in comparison to the uninjured eye from images in (G). Each point represents 1 animal. Ratios are shown as a percentage. Error bars are 1 standard deviation. Statistical significance was determined with two-tailed unequal variance student’s *t*-test. *p<0.05, **p<0.01, ***p<0.001. Scale bars are 100 μm.

The planarian has an innate ability to sense injury size by recognizing the amount of tissue removed, known as the “missing tissue response” or “size sensing mechanism” [38]. The brain and pharynx are planarian organs that are particularly stereotyped in their remodeling following injuries and can also scale dynamically [15,16,39]. For example, in relation to the amount of tissue removed or amputated, the post-mitotic planarian pharynx must be rescaled and re-patterned to its new fragment size, which is accomplished in part by increases in cell death [39]. To test how this scaling mechanism may be altered in *yki(RNAi)*, pharynxes were chemically amputated from trunk fragments that had been regenerating for 3 days but underwent selective amounts of tissue removal (0%, 10%, 40%, and 80%) (Fig 6A). The isolated pharynxes were immediately fixed and assayed for TUNEL (Fig 6B). In *control(RNAi)* amputated pharynxes, a proportionality between the amount of tissue removed and the amount of cell death was observed, as previously reported (Fig 6B) [39]. However, in *yki(RNAi)* animals, this relationship was uncoupled (Fig 6B and 6C).

Similar to the pharynx, the planarian brain must be scaled down in size in relation to the new size of the head fragment [10,16]. Using DAPI and *chat* (*choline acetyltransferase)*, which labels majority of the central brain neurons, a ratio of the brain size relative to the body size was determined (Fig 6D) [40]. At 14 dpa, *yki(RNAi)* head fragments were smaller due to inability to regenerate [22], however, the brain to body ratio was significantly larger (Fig 6D). Accordingly, *cintillo*, which is expressed in chemoreceptive neurons, did not re-scale appropriately in *yki(RNAi)* animals (Fig 6D) [16,41]. Other scaling defects in *yki(RNAi)* animals at 14 dpa included: the *slit* midline gradient in all three regenerating fragments (Fig 6E) [42], *noggin-7* and *tolloid* (S6A Fig) [43,44], and the gut by *HNF4* expression (S6B and S6C Fig) [8]. In total, we concluded from these data that Yki was required for correct tissue remodeling in order to restore scale and proportion of multiple organ systems following injury.

**Fig 7:**
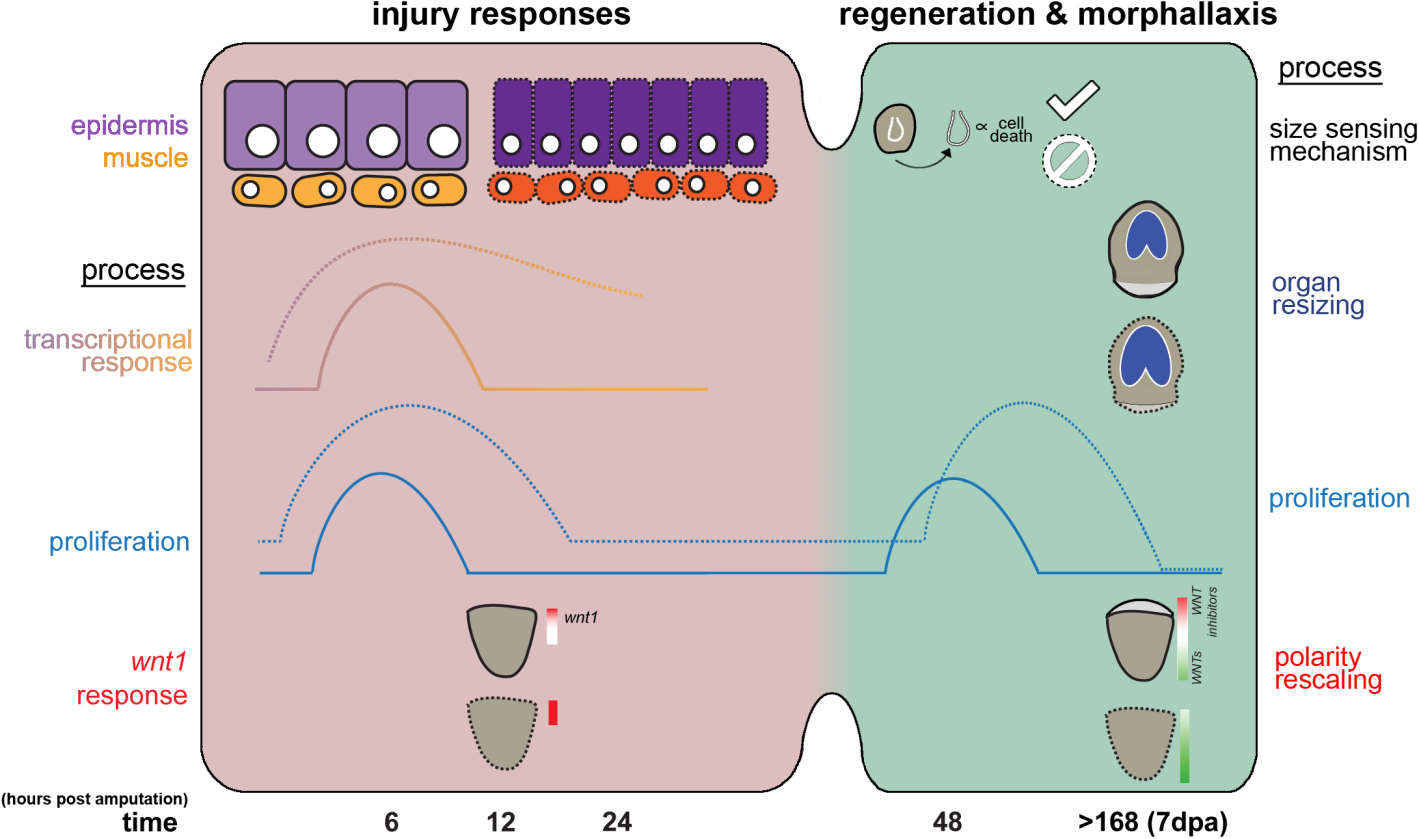
Model: The roles for *yki* during planarian regeneration. In *control(RNAi)* animals (solid lines), the injury responses are composed of distinct spatial and temporal patterns of proliferation and transcription. For instance, the transcriptional response of wound-induced genes is primarily localized to the epidermis and muscle, while the *wnt1* response peaks at 12 hpa. As regeneration progresses, a second wave of proliferation occurs at 48 hpa. After the lost tissues have been replaced, reintegration with the old tissues occurs by morphallaxis. For example, the brain will be rescaled down in size in head fragments, and the WNT genes re-establish their graded expression along the anterior-posterior axis. By contrast, in *yki(RNAi)* regenerating animals (dotted lines), all aspects of the generic wound response are heightened, evident by dysregulated proliferative dynamics and the augmented wound-induced gene expression, which may be attributed to the increased epithelial and muscle cell numbers. The second mitotic burst is temporally shifted and a complete failure in regeneration is observed. Ultimately, re-patterning and scaling are also affected. Altogether, this suggests that *yki* is required to restrict multiple aspects the injury response, including proliferation, wound-induced gene expression, and patterning to ensure proper regenerative outcomes.

It was recently shown that if a planarian eye is scratched off, it will regenerate via a homeostatic mechanism that is not at the threshold to trigger the injury response [45]. However, if the eye is scratched off together with lateral injuries, the eye will regenerate faster because an injury threshold was reached [45]. Because *yki(RNAi)* animals have heightened injury responses, even in intact animals (S7 Fig), we reasoned that an eye scratch alone could reach an injury threshold and predicted that *yki(RNAi)* eyes would reappear faster. We confirmed this prediction and not only observed that *fos-1* and *delta-1* were upregulated following an eye scratch, but by day 12, the regenerating eye in *yki(RNAi)* animals was significantly larger than control (Fig 6F–6H). Interestingly, the growth endpoint of the regenerating eyes in *yki(RNAi)* animals did not end at the correct scale and proportion, and instead grew back significantly larger than controls at 50 dpa (Fig 6G–6H). Taken together, this suggested that *yki* was required to determine organ scaling by restricting the magnitude and duration of injury responses (Fig 7).

## Discussion

### A broad role for yki in wound response?

Our previous work with *yki* in planarians was primarily on the pleiotropic roles in intact animals. We described additional defects in regeneration, but did not have tools or markers to explore those defects. We had demonstrated that cell division was not blocked, nor was differentiation [22]. Here, we focused on why regeneration was failing in *yki(RNAi)* fragments. It was not particularly surprising that *yki(RNAi)* animals displayed hyper-proliferation, accompanying increased differentiation, and increased TUNEL staining, similar to Yki’s role in intact animals. More interesting was the up-regulation of key members of the transcriptional wound response (summarized in Fig 7).

In the broad context of phenotypes for mutants in the Hippo pathway, it is well known that this gene cascade controls tissue growth, patterning, proliferation, and cell death. It would be interesting to determine whether specific tissue defects in other systems are caused by dysregulation of wounding programs. For example, mutations in TAZ, the vertebrate paralog of YAP, cause polycystic kidneys [46,47] and cystic kidneys also have altered Hippo signalling [48], while mutations in the core kinase cassette of the Hippo pathway in adults can cause tissue overgrowths in specific instances of homeostasis or regeneration [49]. In planarian regeneration, we find that both the wound response (Fig 2 and Fig 3), as well as patterning molecules, are dysregulated (Fig 5H). If both responses are under the control of Yki/YAP in other systems, it could explain many of the disparate phenotypes observed in various mutants in the Hippo pathway.

### Direct or indirect effects of yki on the wound response?

A lingering question from this work is whether Yki is a direct repressor of the transcriptional wound response, or whether it is a secondary defect in other tissue types. Although Yorkie/YAP is commonly associated with gene activation, it may also function as a co-repressor by associating with the NuRD complex to repress downstream transcription [50]. Alternatively, it has been well established that both the epidermis and muscle of planarians are the sites of the transcriptional wound response, to a large extent [12,13], and these very tissues are increased in *yki(RNAi)* animals (Fig 4 and Fig 5). Because intact *yki(RNAi)* animals show increased epithelial differentiation and increased muscle cells prior to amputation (S7A and S7B Fig), perhaps an alternative model is that there are simply more cells to transcribe a particular wound-responsive gene or that defects in the epithelium causes induction of wounding, similar to what was observed in [30]. In this case, Yki’s role would indirectly affect the wound response and instead, Yki may function in establishing the appropriate cell size and cell density ratio of a given tissue, such as the epidermis. Although we cannot rule out this explanation due to the pleiotropy of Yki, we believe that Yki’s effects on the wound-response in planarians is direct due to the fact that we detect increased expression of key genes in the wound program in intact animals prior to any injury (S7C and S7D Fig).

### Yki’s cellular role in planarian regeneration: cell autonomous or non-autonomous?

Although Yki is thought to be a transcriptional co-activator, there are clearly cell non-autonomous effects in *yki(RNAi)* animals. For example, proliferation is altered in stem cells, yet *yki* is not expressed appreciably in the stem cell compartment. Because stem cell proliferation in planarians has stereotyped responses to injury and the injury program is increased in *yki(RNAi)* animals (Fig 2 and Fig 3), we suggest that the simplest explanation of the data is that *yki(RNAi)* causes de-repression of the wound program, thereby increasing the stem cell response through extrinsic signals. Although the wound-induced signal(s) that directly trigger the bimodal waves of proliferation during regeneration remain elusive in planarians, correlations between the transcriptional and proliferative response may be inferred. For example, the two responses overlap temporally [11,12,13] and *follistatin(RNAi)* regenerating animals fail to express a second wave of wound-induced genes and do not mount a second burst of proliferation [38,51]. Therefore, the transcriptional injury response, which is regulated by Yki (Fig 2 and Fig 3) may be required to initiate proliferation. Similarly, during *Drosophila* midgut regeneration, Yki is activated in the non-proliferative enterocytes to secrete cytokines that stimulate intestinal stem cell proliferation in a non-autonomous fashion [52,53].

Equally possible is that Yki has roles in cell death or cell longevity, which can also explain the defects seen in *yki(RNAi)* animals. For example, the increased epithelial cells in *yki(RNAi)* could be due to either increased differentiation, or increased longevity of the epithelial cells themselves. A similar mechanism could be at work for muscle since both the epithelium and muscle express *yki* (S1D Fig). Finally, we observe consistently increased cell death in *yki(RNAi)* animals (S1C Fig). In other systems, dying cells often express cytokines to trigger their replacement [54,55]. Although we have no evidence for this compensatory-proliferation mechanism of signaling in planarians, it could be a conserved mechanism of triggering increased cell divisions, thereby leading to increases of specific cell types. A key reagent to distinguish these possibilities would be an antibody and to determine what cell types and in what contexts Yki becomes activated and goes into the nucleus.

### Does yki function in tissue scaling in planarians?

Another key feature of the transcriptional injury response is to lay the foundation for axial and tissue re-scaling, yet the biology of tissue scaling in planarians remains nebulous. The 12 hpa time point is the peak temporal expression window for the majority of wound-induced patterning molecules [12,13], including *wnt1*, which is crucial in determining axial polarity [35]. Interestingly, many of the dysregulated wound-induced genes in *yki(RNAi)* have roles in patterning (Fig 3A and Fig 5H). Indeed, *wnt1* was significantly increased in *yki(RNAi)* animals (Fig 5E). Modulating patterning can have consequences in regeneration by affecting pole determination, cell fate decisions, or organ sizing and scaling [15,16,33,56,57,58]. We hypothesize that the failures in regeneration in *yki(RNAi)* may be attributed to the mis-expression of multiple patterning molecules (Fig 5H). In addition, the process of morphallaxis was also altered in *yki(RNAi)* (Fig 6D and 6E, S6 Fig). Therefore, the dysregulated transcriptional injury response in *yki(RNAi)* may be responsible for the changes in proliferation and patterning that likely have detrimental effects on scaling and sizing during regeneration (Fig 6F and 6H and Fig 7).

What could be causing the upregulation of the transcriptional injury response in *yki(RNAi)* animals? One possibility may be epithelial integrity which serves as a negative feedback regulator of the transcriptional injury responses. *Smed-egr-5(RNAi)* (*early growth factor 5*), a crucial post-mitotic epidermal determinant, causes a loss of epithelial integrity and increased wound-induced gene expression [30]. Similarly, *yki(RNAi)* animals had increased epidermal density concurrent with increased wound-induced gene expression (Fig 4). In other systems, the roles for YAP in regulating cell density converge upon inputs such as cytoskeletal tension and mechanosensation [59,60]; yet, how tension may function in planarian biology, or in a regenerative context, remains unknown. Another source of upstream cues may come from the previously characterized pathways that have roles in regulating the planarian injury responses, including TGFβ signalling through the Activin-Follistatin axis, SMG-1, mTOR, and JNK signalling [38,61,62]. Yki/YAP also have described roles in interacting with each of these pathways, however, the interactions remain to be biochemically elucidated in planarians [63,64,65,66]. Therefore, multiple wounding cues and signals may converge on Yki to direct the injury responses. Taken together, we have shown that Yki is required to restrict the magnitude and duration of the injury responses, which coordinate proliferation, differentiation, and patterning to ultimately dictate scaling during regeneration.

## Materials and methods

### Animal husbandry, exposure to γ-irradiation, and RNAi

Asexual *Schmidtea mediterranea* strain CIW4 were reared as previously described [67]. For irradiation experiments, planarians were exposed to 60 Gray (Gy) of γ-irradiation from a ^137^Cs source [68]. RNAi experiments were performed using previously described expression constructs and HT115 bacteria [69]. Bacteria were grown to an O.D.600 of 0.8 and induced with 1 mM IPTG for 2 hours. Bacteria were pelleted, mixed with liver paste at a ratio of 500 μl of liver to 100 ml of original culture volume, and frozen as aliquots. The negative control, “*control(RNAi)*”, was the *gfp* sequence as previously described [70]. All RNAi food was fed to one week starved experimental worms every 3^rd^ day for a total of 3 feedings. To suppress edema formation, animals were placed in a high-salt medium following RNAi with increasing titrations of 10 mM Instant Ocean Aquarium Salt added into the media every 3 days until a final concentration of 60 mM was achieved [22]. Amputations were performed 15 days after the final feeding with the corresponding salt addition mentioned, which did not alter regeneration or worm behaviour. All animals were size-matched between experimental and control worms.

### Pharynx amputations

Pharynxes were chemically amputated by replacing planarian water with 100 mM sodium azide (NaN_3_) diluted in planarian water. After 7–10 minutes of gentle swirling and pipetting, pharynxes were either entirely extruded or extended, which required forceps for complete removal. NaN_3_ was vigorously washed out with planarian media and subsequently fixed [39,71].

### CHX experiments

Cycloheximide (Sigma) was added immediately to animals following amputation as previously described [12].

### Quantitative real-time PCR (qRT-PCR)

Reverse transcription reactions were conducted on total RNA extracted from approximately 15 worms using a SuperScript III Reverse Transcriptase Kit (Invitrogen). Quantitative real-time PCR was performed in biological triplicate on a Bio-Rad CFX96 Touch Real-Tie PCR Detection System with SYBR Green PCR Master Mix (Roche) as per manufacturer’s instructions. Expression was normalized to *control(RNAi)* intacts and the 2^-ΔΔCT^ method was used for relative quantification. Primer pairs for ubiquitously expressed GAPDH were used as a reference as previously described [72]. Experiments were biologically and technically triplicated. All error bars are standard deviations with statistical significances determined by two tailed unequal variance student’s *t*-tests.

### Immunolabeling, BrdU, TUNEL, and in situ hybridizations (ISH)

Whole-mount ISH (WISH), and double fluorescent ISH (dFISH), and immunostainings were performed as previously described [29,73,74]. Colorimetric WISH stains were imaged on a Leica M165 fluorescent dissecting microscope. dFISH and fluorescent phospho-histone H3 (rabbit monoclonal to H3ser10p, 1:500, Millipore) immunostains were imaged on a Leica DMIRE2 inverted fluorescence microscope with a Hamamatsu Back-Thinned EM-CCD camera and spinning disc confocal scan head. BrdU (Sigma B5002-5G, 25 mg/ml) was dissolved in 50% ethanol and fed to animals and was stained as previously described [24]. TUNEL was performed as previous described [39] with the Terminal Deoxynucleotidyl Transferase enzyme (Thermo, EP0162). All cell counts and co-localizations were quantified using freely available ImageJ software (http://rsb.info.nih.gov/ij/) with the cell counter function. Positive cells were visually distinguished manually. Significance was determined by a two tailed unequal variance pairwise student’s *t*-test. All images were post-processed in a similar manner using Adobe Photoshop.

### RNA sequencing and differential expression analysis

RNA deep sequencing (RNAseq) was performed on 1 day post-irradiated animals with no amputation (intact) or tail fragments regenerating at 6, 12, and 24 hours post amputation. Experiments were performed in biological-triplicate, sequenced to a depth of >20 million reads per sample, and multiplexed on an Illumina HiSeq2500 with 50 base pair, single-end reads. Raw scRNAseq data from uninjured cells (including stem cells, neurons, gut, epithelial, muscle and parapharyngeal cells) were obtained from the NCBI Sequence Read Archive (SRA:PRJNA276084) [13]. Reads were aligned to the SmedASXL transcriptome assembly under NCBI BioProject PRJNA215411 using bowtie2 [75] with 15 bp 3’ trimming. For detecting novel wound-induced genes, DEseq2 was used for differential expression analysis on triplicated regenerating samples at each time point compared to intact animals with [FC] (fold change) ≥ 2 with a [FDR] (false discovery rate) < 0.05[76]. To uncover and validate whether the novel-wound genes were upregulated in *yki(RNAi)* tail fragments, DEseq2 was used to compared time matched samples in a pair-wise manner with significance calling at [FC] ≥ 1.25 and a [FDR] < 0.05 (S1 Table). Tail enrichment was determined with previously published data sets [29]. To detect mis-expressed patterning molecules (Fig 5H), DEseq2 was run on the triplicated *yki(RNAi)* samples and the matched *control(RNAi)* with [FC] ≥ 1.2 or [FC] <0.83 with a [FDR] < 0.05. Venn diagrams were generated on http://www.biovenn.nl/ [77]. Violin plots were produced using modified source code from [78] and heatmaps were produced using the modified heatmap.3 source code from [27]. Heatmaps in Fig 3A and S4A and S4B Fig have expression levels row-normalized to *control(RNAi)* intacts for ease of visualization. All raw RNAseq data and DEseq2 outputs from this manuscript are available at the NCBI Gene Expression Omnibus (GEO) project GSE97787.

## Supporting information

S1 Fig Aberrant proliferative dynamics are observed in *yki(RNAi)* tail fragments even though *yki* is not highly expressed in the stem cell population. (A) The percentage of mitoses occurring in each zone was determined by dividing tail fragments at 48 and 72 hpa into 5 equal compartments (20% each) with zone 1 at the anterior, closest to the wound site (schematic). Representative binning images are shown with red lines demarking each zone and yellow dotted lines outlining the animal (n≥7). Quantifications of the number of mitoses in each zone relative to the total amount are on the right. (B) Tail fragments at 6 hpa were assayed for H3P (red), *piwi-1* (blue), and sigma stem cell marker *soxP-2* (green) with quantification on the right. (C) 100 μm from the wound margin (red dotted line), an increase in both proliferation (green) and apoptosis (magenta) are seen in *yki(RNAi)* animals with quantification on the right (n≥11). (D) From single-cell RNAseq, *yki* expression is enriched in the differentiated tissues (epidermal, gut, and muscle), and is lowly expressed in the stem cell compartment. Error bars are standard deviation. Statistical significance was determined with two-tailed unpaired student’s *t*-test. n.s. = not significant, ***p<0.001. Scale bars are 100 μm.
(TIF)

S2 Fig *yki* restricts wound induced genes. (A) A regeneration time course with representative WISH images for injury marker *tyrosine-kinase2*. (B-C) Cyclohexamide (CHX) does not affect *fos-1* or *delta-1* (B) but abolishes *noggin-like-1* expression (C).
(TIF)

S3 Fig Loss of protonephridial or anterior-posterior maintenance does not affect proliferation or wound-induced gene expression. (A) Knockdown of *yki*, *carbonic anhydrase VII* (*CAVII*), *pou2/3*, and *wnt1* are verified by WISH. Black arrow indicates *wnt1* expression with magnified panel to the right. Besides *yki(RNAi)*, regenerating tail fragments for the other RNAi conditions do not show a regeneration defect (B), changes in proliferation (C) or changes in *fos-1* (D) or *delta-1* expression (E).
(TIF)

S4 Fig *yki* affects wound-induced genes that predominantly localize to the epidermis and muscle. (A) A heatmap of previously identified wound-induced genes (Wurtzel et al., 2015) that were upregulated in our analyses when comparing *control(RNAi)* regenerating tails to *control(RNAi)* intact animals. Red asterisks indicate transcripts that were also significantly up in *yki(RNAi)* regenerating tails at any time point. (B) A heat map of upregulated novel wound-induced genes comparing *yki(RNAi)* tails to *control(RNAi)* tails that are tail-enriched (top) or not (bottom). (C) A representative regeneration time course stained by WISH (left) with corresponding CPM values for *SmedASXL_061347*. Statistical significance was determined by DEseq2 analyses with ***p<0.001. Error bars are standard deviation. Scale bars are 100 μm. (D) From scRNAseq from Wurtzel et al. (2015), differentiated cell type enrichment profiles for novel wound-induced genes that are tail enriched: *Smed-Post-2d* and *beta-catenin-like*; or not: *IMDH2*, *CC122*, *F6QPC8*, *collagen-alpha-5*, and *SmedASXL_061347*.
(TIF)

S5 Fig Wound- and non-wound-induced patterning molecules are improperly expressed during the early injury time points in *yki(RNAi)*. (A-D) A WISH regeneration time course with representative images for *notum* (A), *sfrp-2* (B), *netrin-2A* (C) and *slit* (D). Blue arrows indicate area where *netrin-2A* expression is most prominently wound-induced. Scale bars are 100 μm.
(TIF)

S6 Fig Scaling of patterning gradients and organs are affected in *yki(RNAi)* animals. (A) Representative images of 14 dpa regenerating fragments assayed for *noggin-7* and *tolloid* by WISH. (B) Trunk fragments at 14 dpa are assayed by FISH for gut marker *HNF4* (magenta). (C) Quantification of the area of *HNF4* expression to the total body size from images in (B). Error bars are standard deviation and statistical significance was determined with two-tailed unpaired student’s *t*-test with *p<0.05, ***p<0.001. Scale bars are 100 μm.
(TIF)

S7 Fig *yki(RNAi)* intact animals show increased numbers of muscle and epidermal cells with elevated levels of wound marker expression. (A) Animals were assayed by WISH for *prog-2*, *AGAT-1*, and *collagen*. (B) Quantification of images from (A). (C-E) Wound markers are elevated in *yki(RNAi)* animals, but are still induced during regeneration. Animals were assayed by WISH for wound markers *fos-1*, *delta-1*, and *jun-1* (C). Fold change of *bona fide* wound markers (list from S4A) between *yki(RNAi)* intacts to *control(RNAi)* intacts (D). The same set of wound markers from (E) comparing *yki(RNAi)* regenerating tails to *yki(RNAi)* intacts. Therefore, in *yki(RNAi)* intacts, wounding genes are elevated, but the majority are still induced following injury, which suggests that *yki(RNAi)* animals are still competent to respond to injuries. Error bars are standard deviation and statistical significance was determined with two-tailed unpaired student’s *t*-test with *p<0.05, ***p<0.001. Scale bars are 100 μm.
(TIF)

S1 Table Gene lists and novel genes upregulated in *yki(RNAi)* tails from Fig 3 and S4 Fig. (XLSX)

S2 Table Significantly dysregulated patterning genes in *yki(RNAi)* tails from Fig 5H. (XLSX)
