# Supplementary figures and images for: Yorkie is required to restrict the injury responses in planarians
Source: PLoS Genet. 2017 Jul 7;13(7):e1006874. doi: 10.1371/journal.pgen.1006874 (PMC5515462; doi:10.1371/journal.pgen.1006874)

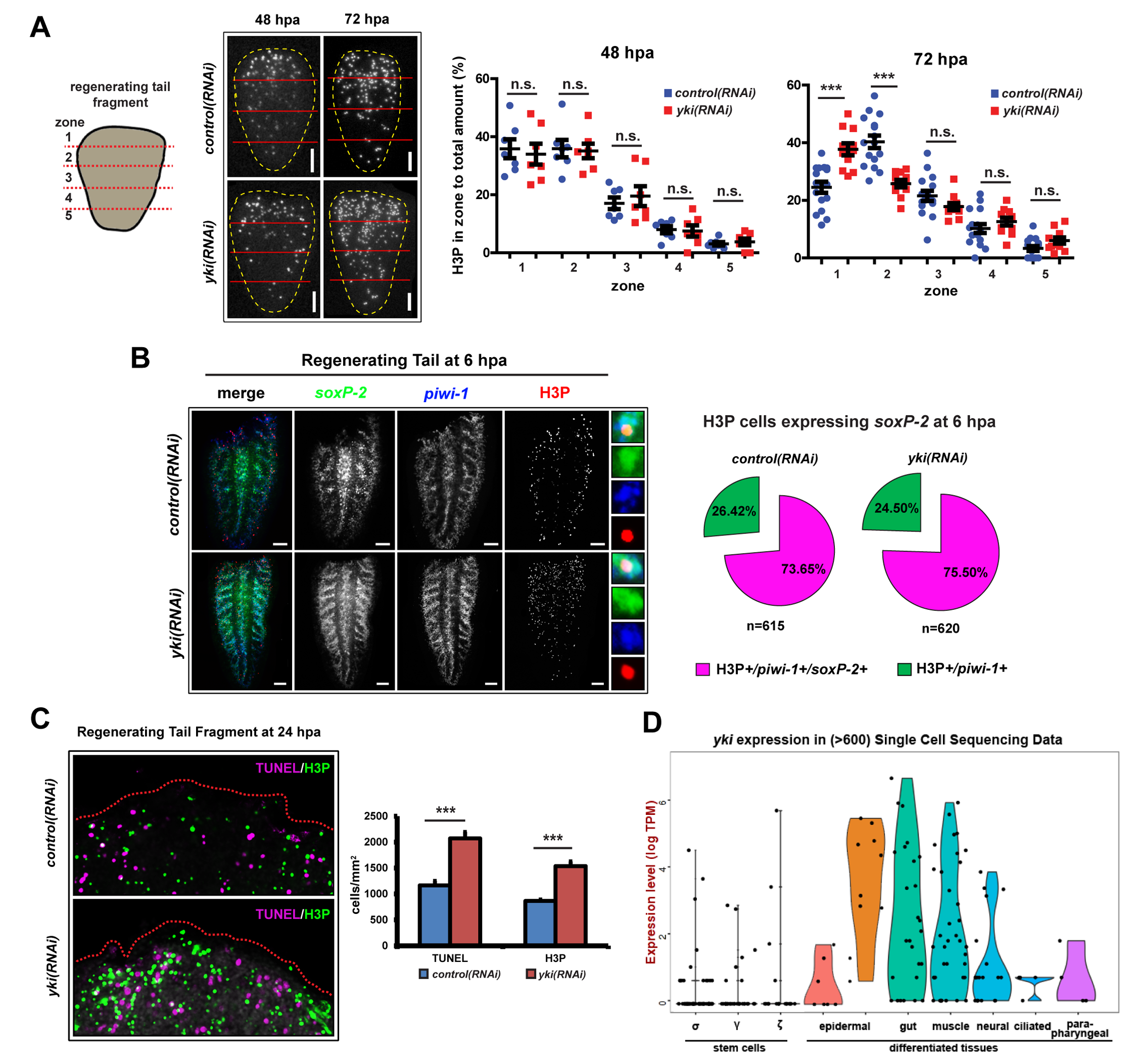

Supplement: S1 Fig — (A) The percentage of mitoses occurring in each zone was determined by dividing tail fragments at 48 and 72 hpa into 5 equal compartments (20% each) with zone 1 at the anterior, closest to the wound site (schematic). Representative binning images are shown with red lines demarking each zone and yellow dotted lines outlining the animal (n≥7). Quantifications of the number of mitoses in each zone relative to the total amount are on the right. (B) Tail fragments at 6 hpa were assayed for H3P (red), piwi-1 (blue), and sigma stem cell marker soxP-2 (green) with quantification on the right. (C) 100 μm from the wound margin (red dotted line), an increase in both proliferation (green) and apoptosis (magenta) are seen in yki(RNAi) animals with quantification on the right (n≥11). (D) From single-cell RNAseq, yki expression is enriched in the differentiated tissues (epidermal, gut, and muscle), and is lowly expressed in the stem cell compartment. Error bars are standard deviation. Statistical significance was determined with two-tailed unpaired student’s t-test. n.s. = not significant, ***p<0.001. Scale bars are 100 μm. (TIF) [file pgen.1006874.s001.tif]

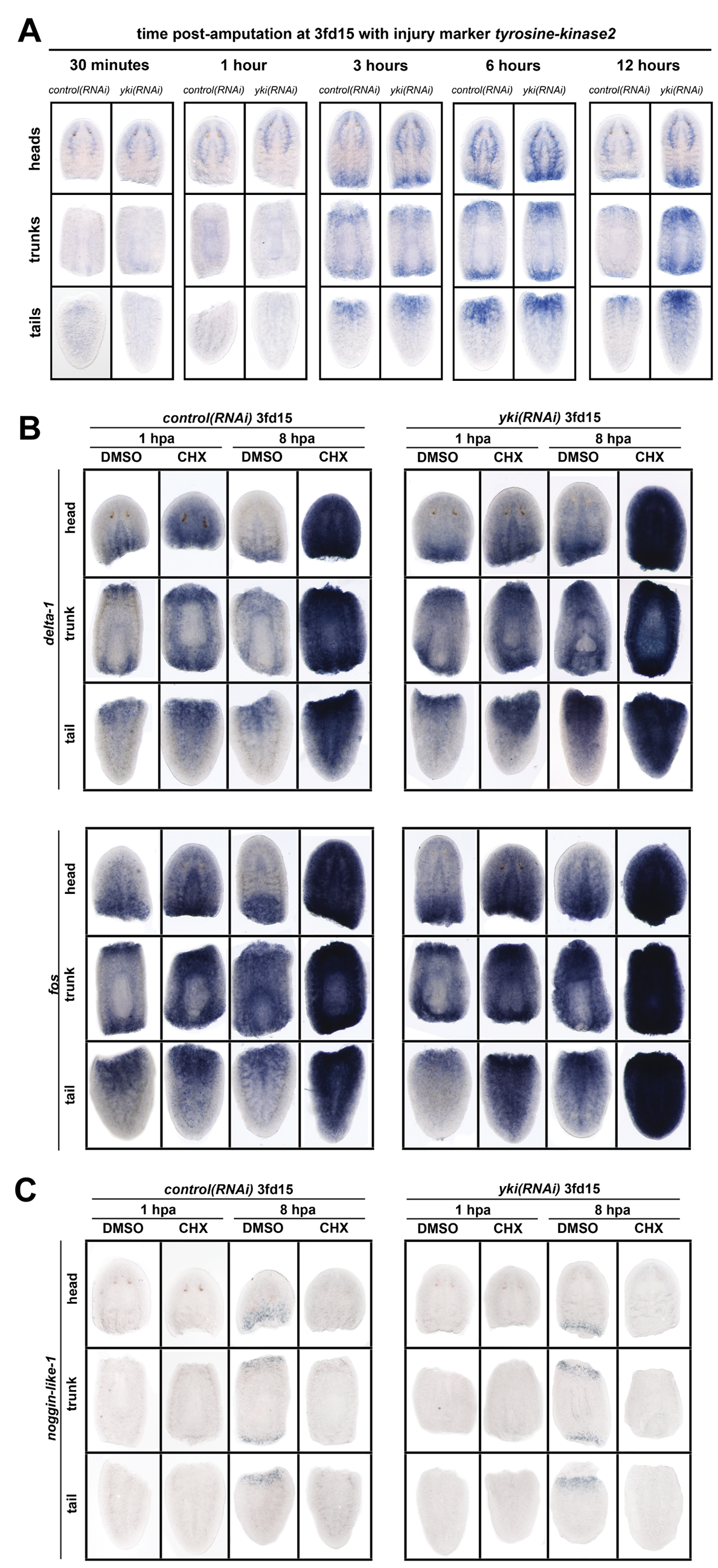

Supplement: S2 Fig — (A) A regeneration time course with representative WISH images for injury marker tyrosine-kinase2. (B-C) Cyclohexamide (CHX) does not affect fos-1 or delta-1 (B) but abolishes noggin-like-1 expression (C). (TIF) [file pgen.1006874.s002.tif]

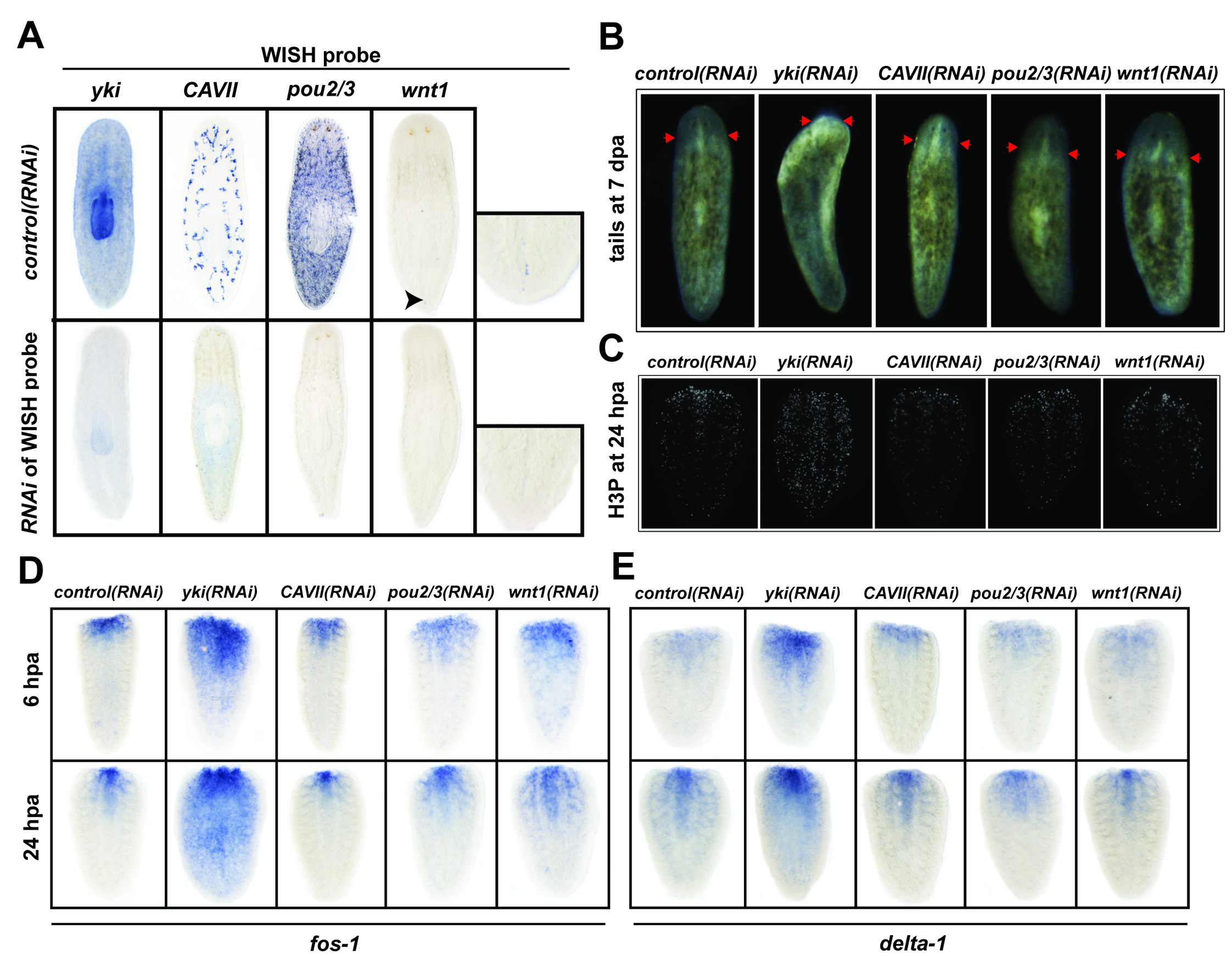

Supplement: S3 Fig — (A) Knockdown of yki, carbonic anhydrase VII (CAVII), pou2/3, and wnt1 are verified by WISH. Black arrow indicates wnt1 expression with magnified panel to the right. Besides yki(RNAi), regenerating tail fragments for the other RNAi conditions do not show a regeneration defect (B), changes in proliferation (C) or changes in fos-1 (D) or delta-1 expression (E). (TIF) [file pgen.1006874.s003.tif]

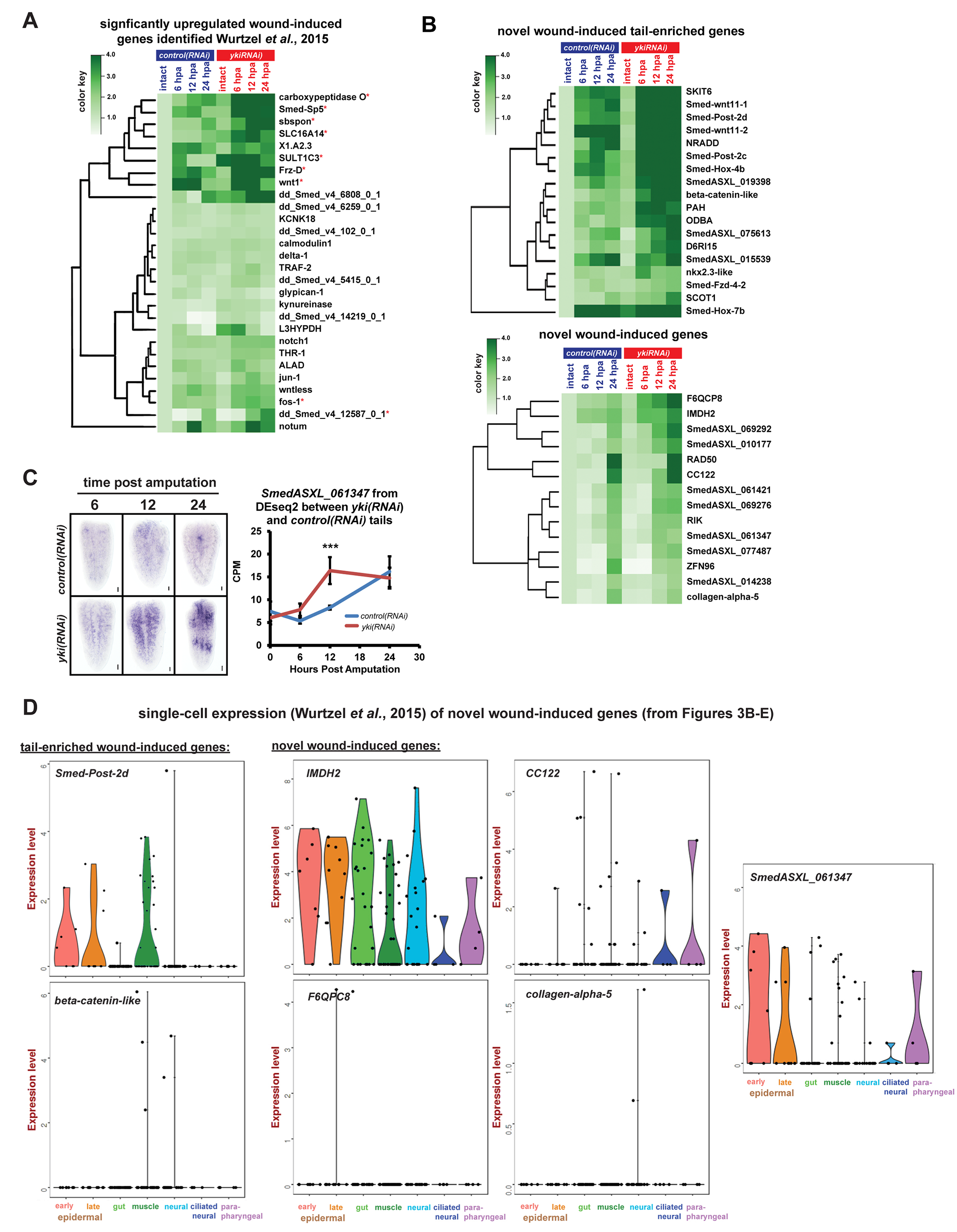

Supplement: S4 Fig — (A) A heatmap of previously identified wound-induced genes (Wurtzel et al., 2015) that were upregulated in our analyses when comparing control(RNAi) regenerating tails to control(RNAi) intact animals. Red asterisks indicate transcripts that were also significantly up in yki(RNAi) regenerating tails at any time point. (B) A heat map of upregulated novel wound-induced genes comparing yki(RNAi) tails to control(RNAi) tails that are tail-enriched (top) or not (bottom). (C) A representative regeneration time course stained by WISH (left) with corresponding CPM values for SmedASXL_061347. Statistical significance was determined by DEseq2 analyses with ***p<0.001. Error bars are standard deviation. Scale bars are 100 μm. (D) From scRNAseq from Wurtzel et al. (2015), differentiated cell type enrichment profiles for novel wound-induced genes that are tail enriched: Smed-Post-2d and beta-catenin-like; or not: IMDH2, CC122, F6QPC8, collagen-alpha-5, and SmedASXL_061347. (TIF) [file pgen.1006874.s004.tif]

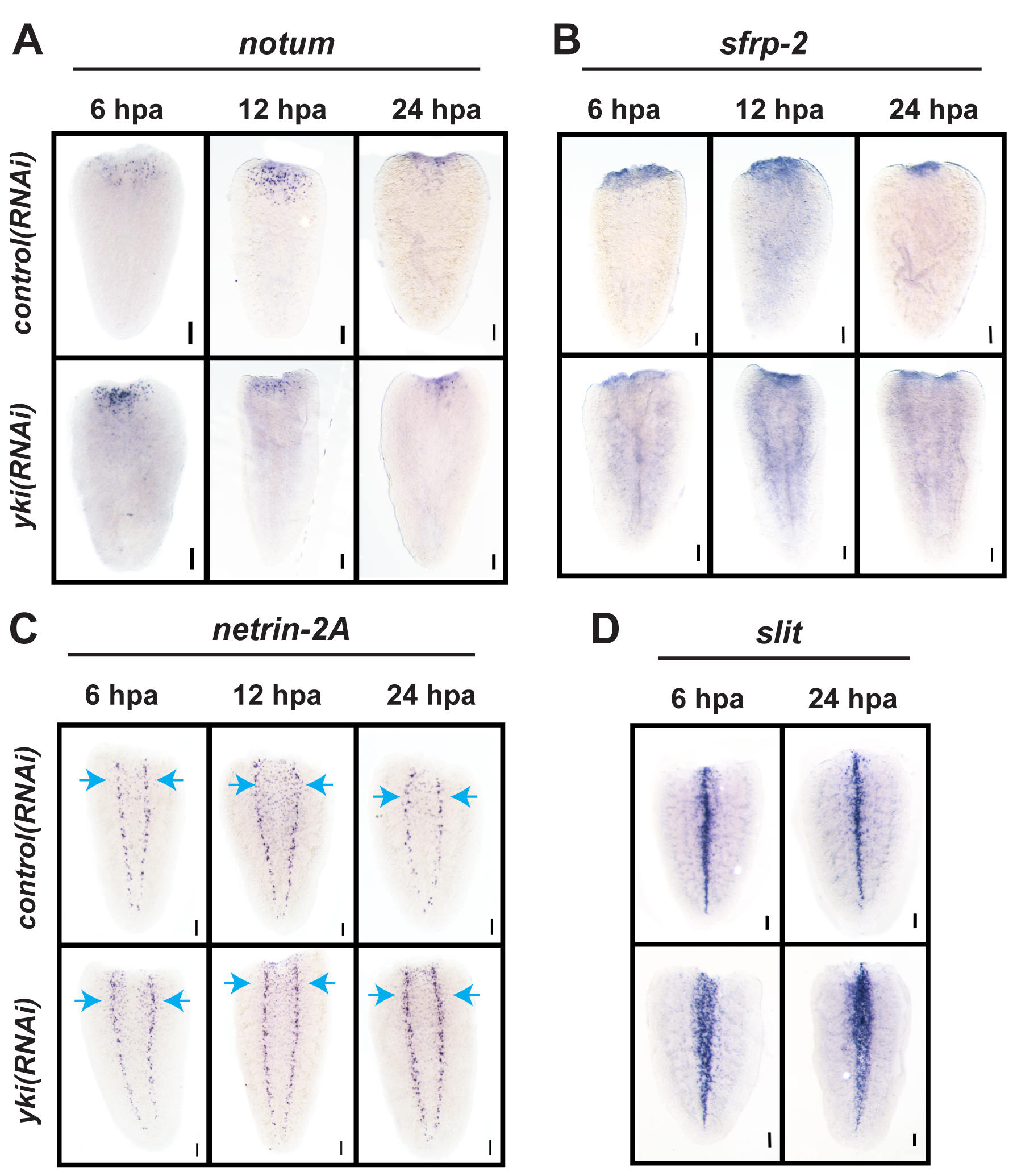

Supplement: S5 Fig — (A-D) A WISH regeneration time course with representative images for notum (A), sfrp-2 (B), netrin-2A (C) and slit (D). Blue arrows indicate area where netrin-2A expression is most prominently wound-induced. Scale bars are 100 μm. (TIF) [file pgen.1006874.s005.tif]

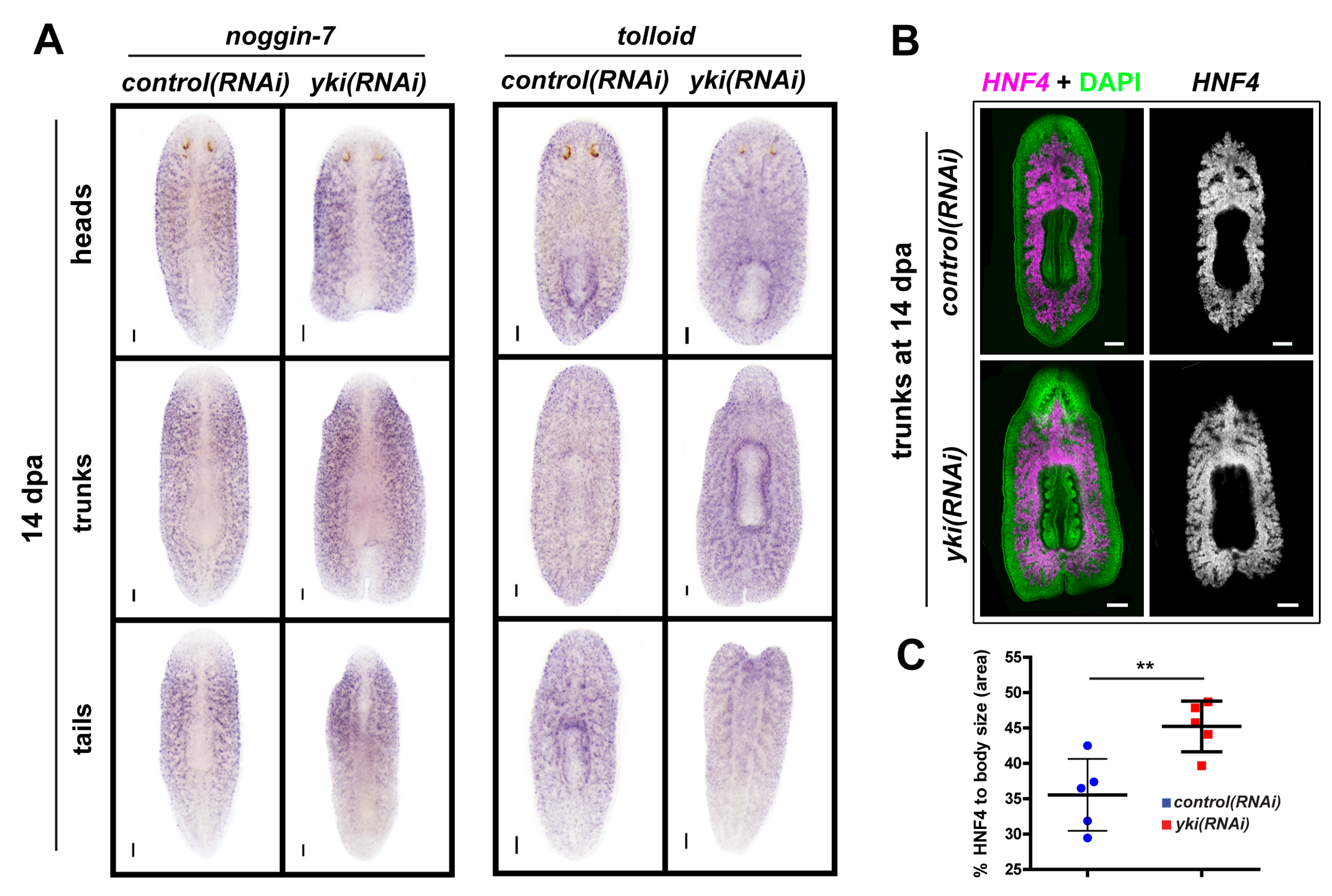

Supplement: S6 Fig — (A) Representative images of 14 dpa regenerating fragments assayed for noggin-7 and tolloid by WISH. (B) Trunk fragments at 14 dpa are assayed by FISH for gut marker HNF4 (magenta). (C) Quantification of the area of HNF4 expression to the total body size from images in (B). Error bars are standard deviation and statistical significance was determined with two-tailed unpaired student’s t-test with *p<0.05, ***p<0.001. Scale bars are 100 μm. (TIF) [file pgen.1006874.s006.tif]

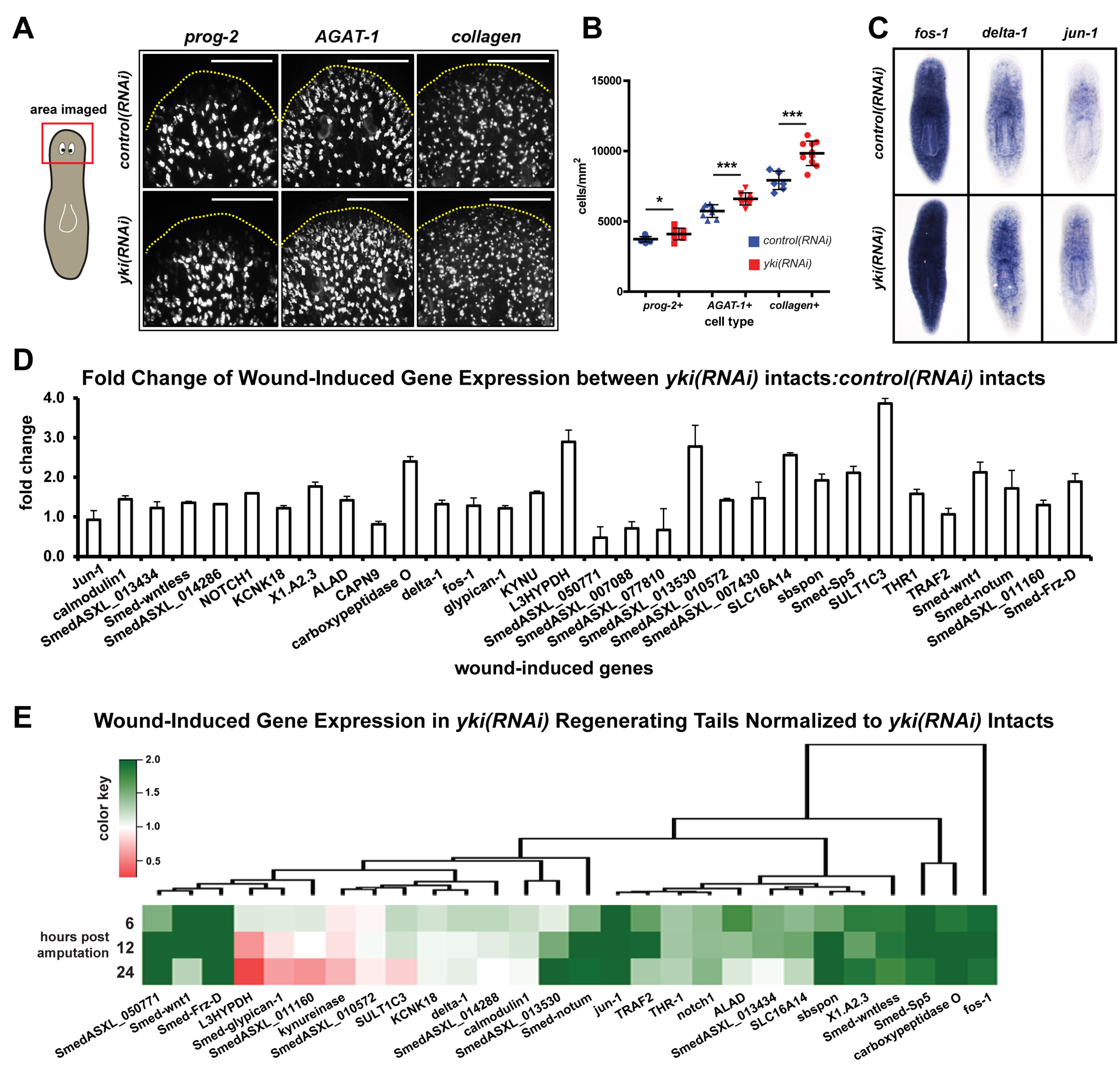

Supplement: S7 Fig — (A) Animals were assayed by WISH for prog-2, AGAT-1, and collagen. (B) Quantification of images from (A). (C-E) Wound markers are elevated in yki(RNAi) animals, but are still induced during regeneration. Animals were assayed by WISH for wound markers fos-1, delta-1, and jun-1 (C). Fold change of bona fide wound markers (list from S4A) between yki(RNAi) intacts to control(RNAi) intacts (D). The same set of wound markers from (E) comparing yki(RNAi) regenerating tails to yki(RNAi) intacts. Therefore, in yki(RNAi) intacts, wounding genes are elevated, but the majority are still induced following injury, which suggests that yki(RNAi) animals are still competent to respond to injuries. Error bars are standard deviation and statistical significance was determined with two-tailed unpaired student’s t-test with *p<0.05, ***p<0.001. Scale bars are 100 μm. (TIF) [file pgen.1006874.s007.tif]
